# Molecular Typing of IberoAmerican *Cryptococcus neoformans* Isolates

**DOI:** 10.3201/eid0902.020246

**Published:** 2003-02

**Authors:** Wieland Meyer, Alexandra Castañeda, Stuart Jackson, Matthew Huynh, Elizabeth Castañeda, the IberoAmerican Cryptococcal Study Group

**Affiliations:** * University of Sydney, Sydney, Australia; † Instituto Nacional de Salud, Bogotá, Colombia; ‡ University of Western Sydney, Campbelltown, Australia

**Keywords:** *Cryptococcus neoformans*, molecular epidemiology, PCR-fingerprinting, *URA5* – RFLP analysis, research

## Abstract

A network was established to acquire basic knowledge of *Cryptococcus neoformans* in IberoAmerican countries. To this effect, 340 clinical, veterinary, and environmental isolates from Argentina, Brazil, Chile, Colombia, Mexico, Peru, Venezuela, Guatemala, and Spain were typed by using M13 polymerase chain reaction-fingerprinting and orotidine monophosphate pyrophosphorylase (*URA5*) gene restriction fragment length polymorphsm analysis with *Hha*I and *Sau*96I in a double digest. Both techniques grouped all isolates into eight previously established molecular types. The majority of the isolates, 68.2% (n=232), were VNI (var. *grubii*, serotype A), which accords with the fact that this variety causes most human cryptococcal infections worldwide. A smaller proportion, 5.6% (n=19), were VNII (var. *grubii*, serotype A); 4.1% (n=14), VNIII (AD hybrid), with 9 isolates having a polymorphism in the *URA5* gene; 1.8% (n=6), VNIV (var. *neoformans*, serotype D); 3.5% (n=12), VGI; 6.2% (n=21), VGII; 9.1% (n=31), VGIII, and 1.5% (n=5) VGIV, with all four VG types containing var. *gattii* serotypes B and C isolates.

Cryptococcosis is among the most prevalent life-threatening mycoses and has a worldwide distribution. The etiologic agent is the basidiomycetous yeast *Cryptococcus neoformans* (*1*,*2*); three varieties are recognized: *C. neoformans* var. *grubii*, serotype A (*3*), *C. neoformans* var. *neoformans,* serotype D, *C. neoformans* var. *gattii*, serotypes B and C, and the hybrid serotype AD (*4*).

Humans are infected by inhaling infectious propagules from the environment, which primarily colonize the lung and subsequently invade the central nervous system (*4*). *C. neoformans* var. *grubii*/*neoformans* has been isolated worldwide from soil enriched with avian excreta (*4*,*5*). More recently, decaying wood from certain species of trees has been proposed as environmental habitat for this variety (*6*). In contrast, the distribution in nature for *C*. *neoformans* var. *gattii* is geographically restricted to mainly tropical and subtropical regions (*7*,*8*). To date, specific host trees are represented by *Eucalyptus* species, *Moquilea tomentosa, Cassia grandis, Ficus microcapra*, and *Terminalia catappa* (*7*–*11*).

Worldwide, in immunocompromised hosts, most infections are caused by *C. neoformans* var. *grubii* (*4*,*5*). In contrast, *C. neoformans* var. *gattii* virtually always affects immunocompetent hosts (*8*).

In the last decade, a number of DNA typing techniques have been used to study the epidemiology of *C. neoformans*. These techniques include karyotyping, random amplification of polymorphic DNA, restriction fragment length polymorphism (RFLP), DNA hybridization studies, amplified fragment length polymorphism (AFLP), and polymerase chain reaction (PCR) fingerprinting (*12*–*17*).

PCR fingerprinting has been used as the major typing technique in the ongoing global molecular epidemiologic survey of *C. neoformans* (*14*,*18*), dividing >400 clinical and environmental isolates into eight major molecular types: VNI (var. *grubii,* serotype A), VNII (var. *grubii,* serotype A), VNIII (serotype AD), VNIV (var. *neoformans*, serotype D), VGI, VGII, VGIII, and VGIV (var. *gattii*, serotypes B and C). No correlation between serotype and molecular type has been found for *C. neoformans* var. *gattii*. The molecular types were recently confirmed by RFLP analysis of the orotidine monophosphate pyrophosphorylase (*URA5*) gene and the phospholipase (*PLB1*) gene (*19*).

Globally, most of the isolates recovered from AIDS patients belong to the genotypes VNI and VNIV, whereas the genotypes VNI and VGI are predominant throughout the world for *C. neoformans* var. *grubii* and *C. neoformans* var. *gattii,* respectively. The larger number of genotype VNI isolates agrees with the fact that *C. neoformans* var. *grubii* causes most human cryptococcal infections worldwide (*18*,*19*).

The aims of this study were the following: 1) to extend the molecular epidemiologic survey to other parts of the world, 2) to establish a regional network of participating reference laboratories, and 3) to apply PCR fingerprinting and *URA5* RFLP typing to investigate the genetic structure and possible epidemiologic relationships between clinical and environmental isolates obtained in Latin America and Spain. The results of this study permitted us to determine the major molecular types and their distribution within each participating country.

## Materials and Methods

### Study Design

During the 12th International Society for Human and Animal Mycoses meeting in Buenos Aires, Argentina, in March 2000, it was decided to establish an IberoAmerican Cryptococcal Study Group under the coordination of E. Castañeda and W. Meyer. Each of the participating laboratories was asked to submit 10–30 isolates. For the clinical isolates, the following data were requested: isolation date, demographic data (age and gender of patient), collection location, risk factors, source and variety, and serotype. For the environmental and veterinary isolates, the data collected included isolation date, source, collection location, variety, and serotype.

### Fungal Isolates

Cryptococcal isolates studied in this study are listed in Appendix Tables 1 and 2. The isolates were obtained by the participating laboratories of the IberoAmerican Cryptococcal Study Group and maintained on Sabouraud dextrose agar slants at 4°C and as water cultures at room temperature. Isolates were identified as *C. neoformans* by using standard methods (*20*). The variety was determined by the color reaction test on L-canavanine-glycine-bromothymol blue medium (*21*), and the serotype was determined, in selected isolates, by the use of the Crypto Check Kit (Iatron Laboratories Inc., Tokyo, Japan).

The isolates were sent for molecular typing to the Molecular Mycology Laboratory at the University of Sydney at Westmead Hospital, Sydney, Australia, either as water cultures or on Sabouraud dextrose agar slants. For long-term storage, the isolates were maintained as glycerol stocks at –70°C.

### Reference Strains

A set of laboratory standard *C. neoformans* reference strains representing each molecular type were used in PCR fingerprinting and *URA5* RFLP as follows: WM 148 (serotype A, VNI), WM 626 (serotype A, VNII), WM 628 (serotype AD, VNIII), WM 629 (serotype D, VNIV), WM 179 (serotype B, VGI), WM 178 (serotype B, VGII), WM 161 (serotype B, VGIII), and WM 779 (serotype C, VGIV) (*14*).

### DNA Extraction

High-molecular-weight DNA was isolated as described previously (*14*). Briefly, *C. neoformans* isolates were grown on Sabouraud’s dextrose agar at 37°C for 48 h, a loopful of cells from the culture was mixed with sterile deionized water and centrifuged. The supernatant was discarded, and the tube containing the yeast cell pellet was frozen in liquid nitrogen. The pellet was ground with a miniature pestle. The cell lysis solution (100 mg triisopropylnapthalene sulfonic acid, 600 mg para-aminosalicylic acid, 10 mL sterile deionized water, 2.5 mL extraction buffer (1 M Tris-HCl, 1.25 M NaCl, 0.25 M EDTA, pH 8.0) and 7.5 mL phenol saturated with Tris-EDTA was preheated to 55°C, and 700 μL of this mixture was added to the frozen, ground cells. The tubes were incubated for 2 min at 55°C, shaken occasionally, and then 500 µL chloroform was added, and the mixture was incubated for a 2 min at 55°C and shaken occasionally. The tubes were centrifuged for 10 min at 14,000 rpm, and the aqueous phase was transferred to a new tube. Then, 500 μL of phenol-chloroform-isoamyl alcohol (25:24:1) was added, shaken for 2 min at room temperature, and centrifuged as above. The aqueous phase was transferred to a new tube, 500 µL of chloroform was added, shaken, and centrifuged as above. To precipitate the genomic DNA, the aqueous phase was again transferred to a new tube, and 0.03 volumes 3.0 M sodium acetate (pH 5.2) and 2.5 volumes cold 96% ethanol were added, and the mixture was gently shaken and incubated at –20°C for at least 1 h or overnight. The solution was centrifuged for 30 min at 14,000 rpm to pellet the DNA. The DNA pellet was washed with 70% ethanol and centrifuged for 10 min at 14,000 rpm and air-dried. The DNA was resuspended in 200 μL sterile deionized water at 4°C overnight and stored at –20°C.

### PCR Fingerprinting

The minisatellite-specific core sequence of the wild-type phage M13 (5′ GAGGGTGGCGGTTCT 3′) (*22*) was used as single primer in the PCR. The amplification reactions were performed in a volume of 50 μL containing 25 ng high-molecular-weight genomic DNA, 10 mM Tris-HCl, pH 8.3, 50 mM KCl, 1.5 mM MgCl, 0.2 mM **[Q1: decimal point OK?]** each of the dATP, dCTP, dGTP and dTTP (Roche Diagnostics GmbH, Mannheim, Mannheim, Germany), 3 mM magnesium acetate, 30 ng primer, and 2.5 U Amplitaq DNA polymerase (Applied Biosystems, Foster City, CA). PCR was performed for 35 cycles in a Perkin-Elmer thermal cycler (model 480) with 20 s of denaturation at 94°C, 1 min annealing at 50°C, and 20 s extension at 72°C, followed by a final extension cycle for 6 min at 72°C. Amplification products were removed, concentrated to approximately 20 μL and separated by electrophoresis on 1.4% agarose gels (stained with ethidium bromide, 10 mg/mL stock) in 1X Tris-borate-EDTA (TBE) buffer at 60 V for 14 cm, and visualized under UV light (*14*). Molecular types (VNI–VNIV and VGI–VGIV) were assigned, according to the major bands in the patterns. All visible bands were included in the analysis, independent of their intensity (*14*,*18*).

### *URA5* Gene RFLP

PCR of the URA5 gene was conducted in a final volume of 50 µL. Each reaction contained 50 ng of DNA, 1X PCR buffer (10 mM Tris-HCl, pH 8.3, 50 mM KCl, 1.5 mM MgCl2; Applied Biosystems, Foster City, CA), 0.2 mM each of dATP, dCTP, dGTP, and dTTP (Roche Diagnostics GmbH), 3 mM magnesium acetate, 1.5 U AmpliTaq DNA polymerase (Applied Biosystems), and 50 ng of each primer URA5 (5'ATGTCCTCCCAAGCCCTCGACTCCG 3') and SJ01 (5'TTAAGACCTCTGAACACCGTACTC 3'). PCR was performed for 35 cycles in a Perkin-Elmer thermal cycler (model 480) at 94°C for 2-min initial denaturation, 45 s of denaturation at 94°C, 1 min annealing at 61°C, and 2-min extension at 72°C, followed by a final extension cycle for 10 min at 72°C. Amplification products were mixed with one fifth volume of loading buffer (15% Ficoll 400, 0.25% orange G, MilliQ water), 15 µL of PCR products were double digested with Sau96I (10 U/µL) and HhaI (20 U/µL) for 3 h or overnight and separated by 3% agarose gel electrophoresis at 100 V for 5 h. RFLP patterns were assigned visually by comparing them with the patterns obtained from the standard strains (VNI-VNIV and VGI-VGIV) (Jackson et al. unpub. data).

### Statistical Analysis

Initially, individual PCR fingerprints were visually compared to those of the standard strains, amplified in parallel, to determine the major molecular type for each isolate. The computer program GelComparII, version 1.01 (Applied Maths, Kortrijk, Belgium) was used to determine the genetic relationship of the strains. DNA bands of each fingerprint pattern were defined manually with a band-position tolerance of 0.9%, being the optimal settings needed to define the molecular size marker bands as 100% identical. Similarity coefficients were calculated by using the Dice algorithm, and cluster analyses were performed by the neighbor-joining algorithms by using the "Fuzzy Logic" and "Area Sensitive" option of the GelcomparII program.

## Results

During the course of this investigation, a network was established with 15 laboratories from nine countries participating in this study. The participant countries were: Argentina, Brazil, Chile, Colombia, Guatemala, Mexico, Peru, Spain, and Venezuela*.*

A total of 340 *C. neoformans* isolates, comprising 266 clinical, 7 veterinary, and 67 environmental isolates were submitted for molecular typing. Of these, 57 were from Argentina (53 clinical and 4 environmental), 66 from Brazil (56 clinical, 9 environmental, and 1 veterinary), 19 from Chile (15 clinical and 4 environmental), 62 from Colombia (39 clinical and 23 environmental), 15 from Guatemala (all clinical), 69 from Mexico (46 clinical and 23 environmental), 13 from Peru (all clinical), 19 from Spain (9 clinical, 6 veterinary, and 4 environmental), and 20 from Venezuela (all clinical). From the total isolates investigated, 271 (79.6%) were *C. neoformans* var. *grubii/neoformans;* 251 (92.6%) of them were *C. neoformans* var. *grubii*, 6 (1.8%) were *C. neoformans* var. *neoformans,* and 13 (4.8%) were AD hybrid isolates. The remaining 69 (20.4%) isolates were *C. neoformans* var. *gattii*.

All 340 isolates were typed by PCR fingerprinting by using the minisatellite-specific oligonucleotide M13 as a single primer and RFLP analysis of the *URA5* gene with the restriction enzymes *Sau*96I and *Hha*I in a double digest. The molecular types were determined for each isolate by comparing the obtained PCR fingerprint profiles and *URA5* RFLP patterns with the respective standard patterns for each molecular type.

The serotyping results (Iatron) correlated with the molecular subtyping results in all serotype B (n=31) and C (n=13) isolates. Regarding serotype A, 99 from a total of 102 (97%) isolates correlated; the remaining 3 were serotype A by the Iatron and serotype AD by the molecular typing method. Regarding serotype D, one of four reported was confirmed by molecular typing; the other three were serotype AD. For serotype AD, two isolates were found when typed with the Iatron kit and eight when typed with molecular typing techniques. All the changes were found in the isolates from Spain. This finding is not surprising, taking into account that problems with the serotyping concerning potential serotype AD hybrids are known (*4*). Appendix Tables 1 and 2 list the characteristics of the studied isolates by participating country, laboratory, laboratory code, clinical, veterinary or environmental origin, isolate characteristics (isolation year, isolation location, source, gender, age and risk factor), variety, serotype and the molecular type identified during this study.

**Figure 1:**
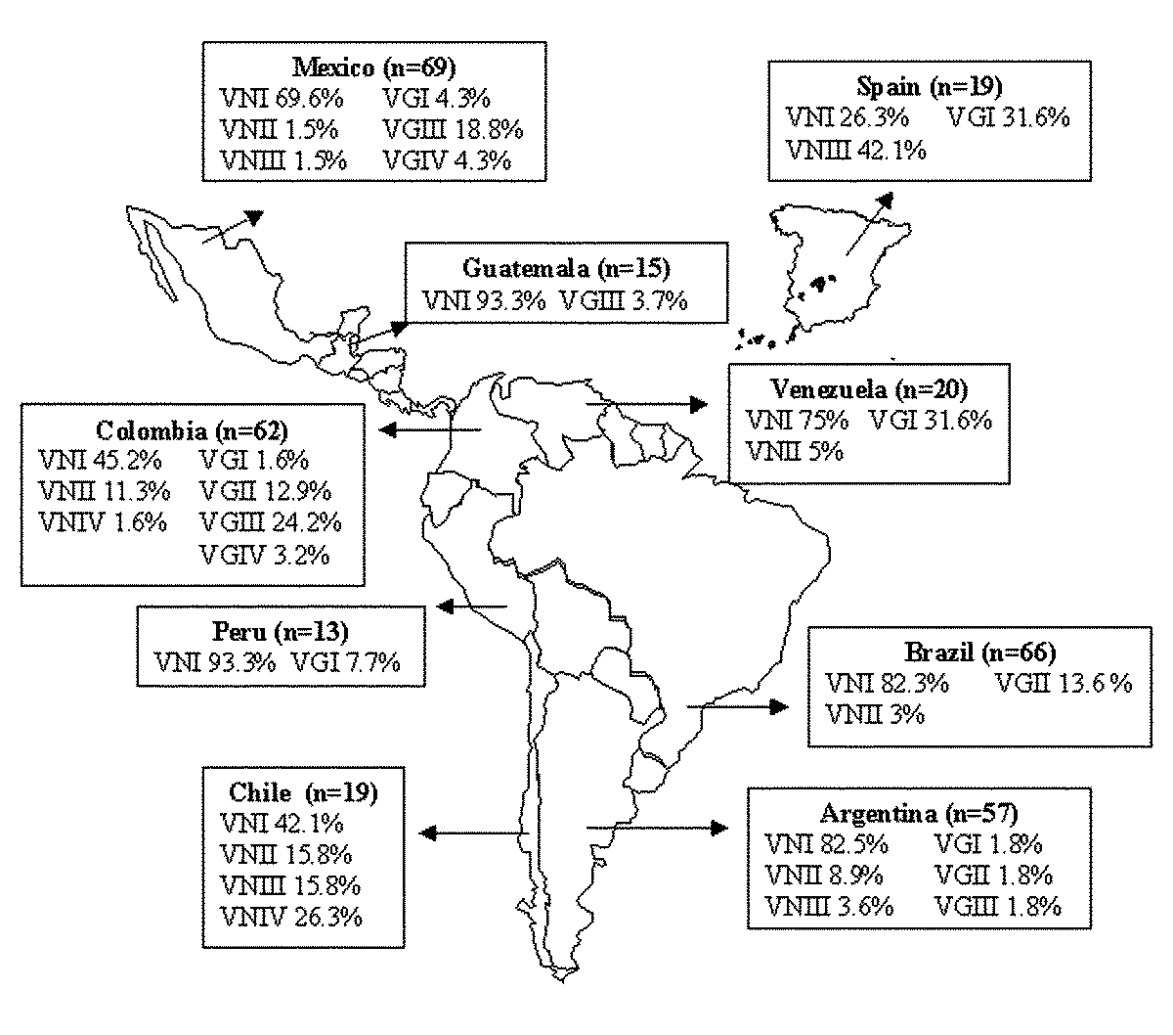
Geographic distribution of the molecular types obtained from IberoAmerican *Crptococcus neoformans* isolates by polymerase chain reaction fingerprinting and *URA5* gene restriction fragment length polymorphis analysis (total numbers studied per country given in parentheses).

**Figure 2:**
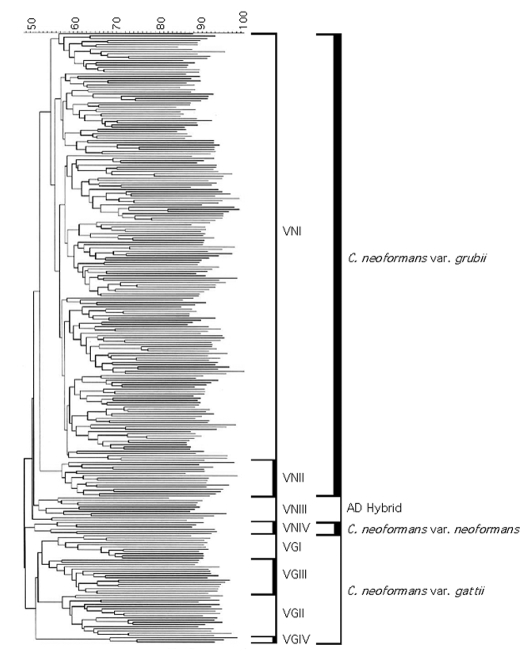
Dendrogram of the polymerase chain reaction-fingerprinting patterns obtained with the primer M13 from a selection of the IberoAmerican isolates studied. All the isolates fall into eight major molecular types, which fall into three major groups corresponding to *Cryptococcus neoformans* var. *grubii*, serotype A, with two molecular types VNI and VNII; *C. neoformans* var. *neoformans*, serotype D, with the molecular type VNIV; and *C. neoformans* var. *gattii* serotypes B and C, with the molecular types VGI, VGII, VGIII and VGIV. In addition to the three major clusters we can see the intermediate molecular type VNIII, representing the AD hybrids.

**Figure 3:**
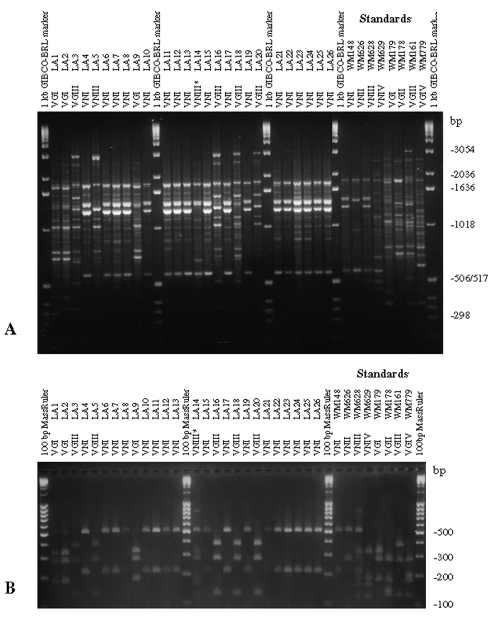
Polymerase chain reaction (PCR) fingerprints generated with the primer M13 (2A), and *URA5* gene restriction fragment length polymorphism (RFLP) profiles identified by double digest of the gene with *Sau*96I and *Hha*I (2B) obtained from a selection of Mexican *Cryptococcus neoformans* isolates, given as a representative example for the patterns obtained from clinical and environmental isolates from Latin America. Standard patterns obtained from the reference strains of the major molecular types by PCR-fingerprinting patterns with the microsatellite specific primer M13 as a single primer in the PCR (right-hand side of 2A) and *URA5* gene RFLP profiles generated after double digest with *Sau*96I and *Hha*I (right-hand side of 2B) (VNIII correspond to the seven-band *URA5* RFLP pattern and VNIII* correspond to the six-band *URA5* RFLP pattern).

**Figure 4:**
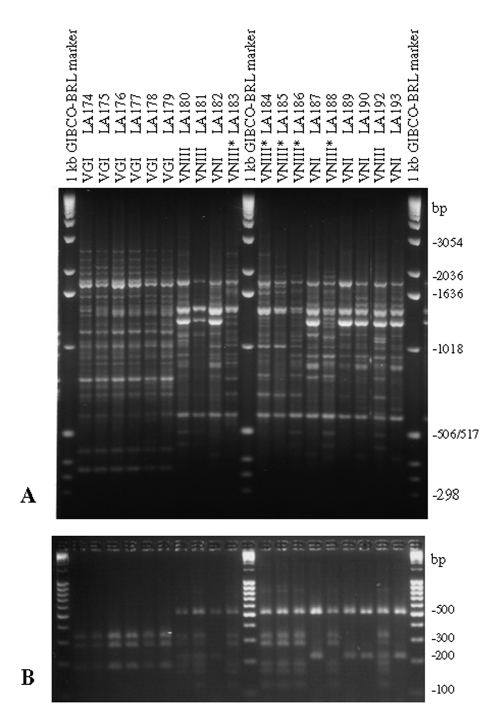
Polymerase chain reaction fingerprints generated with the primer M13 (3A) and *URA5* gene restriction frgement length polymorphism (RFLP) profiles identified via double digest of the gene with *Sau*96I and *Hha*I (3B) obtained from the Spanish clinical, veterinary, and environmental *Cryptococcus neoformans* isolates (VNIII correspond to the seven-band *URA5* RFLP pattern and VNIII* correspond to the six-band *URA5* RFLP pattern).

Both molecular typing techniques grouped the isolates in the eight previously established major genotypes (Figures 1 and 2). From the isolates investigated, 232 (68.2%) were molecular type VNI (serotype A, var. *grubii*), 19 (5.6%) were molecular type VNII (serotype A, var. *grubii*), 14 (4.1%) were molecular type VNIII (serotype A/D, hybrid between the serotypes A and D), with 5 having a RFLP pattern of the *URA5* gene with seven bands, indicated by VNIII in Appendix Tables 1 and 2 and Figure 3B and 4B, corresponding to a hybrid between VNI, VNII, and VNIV and 9 isolates having an RFLP pattern of the *URA5* gene with six bands, indicated by VNIII* in Appendix Tables 1 and 2 and Figure 4B and 5B, corresponding to a hybrid between VNII and VNIV, 6 (1.8%) were molecular type VNIV (serotype D, var. *neoformans*), 12 (3.5%) were molecular type VGI (serotypes B and C, var. *gattii*), 21 (6.2%) were molecular type VGII (serotypes B and C, var. *gattii*), 31 (9.1%) were molecular type VGIII (serotypes B and C, var. *gattii*), and 5 (1.5%) were molecular type VGIV (serotypes B and C, var. *gattii*).

Figures 3A and 4A show examples of PCR fingerprints obtained from Mexican and Spanish isolates; Figures 3B and 4B show the corresponding *URA5* RFLP patterns for the same isolates. The Mexican isolates were selected because they were representative of the patterns observed from all the isolates submitted by the Latin American participating laboratories. The Spanish isolates were selected because they represented a distribution of molecular types corresponding to those observed in previous studies on European isolates (*14*).

From the 340 isolates studied 277, marked with “*” in Appendix Tables 1 and 2, have been included in the GelCompar*II* analysis. Sixty-three isolates were excluded from the analysis since their PCR fingerprinting patterns were not sharp or had been run under slightly different electrophoresis conditions, making the band positions impossible to compare. Cluster analysis of the PCR-fingerprinting profiles by using the GelCompar*II* program grouped all isolates into three major clusters according to variety and into eight major groups according to the molecular type. The overall homology observed was 50.4% among isolates of *C. neoformans* var. *grubii*, 50.9% for *C. neoformans* var. *neoformans,* and 51.2% for *C. neoformans* var. *gattii* (Figure 2). The homology within a given molecular type was as follows: 54.8% VNI, 57.3% VNII, 51.9% VNIII, 50.9% VNIV, 56.4% VGI, 56.4% VGII, 54.4% VGIII, and 68.3% VGIV.

Besides grouping all isolates into the eight major molecular patterns, the molecular type VNI could be subdivided into eight main subclusters, with most of these subclusters’ grouping isolates obtained from specific countries. The similarity between isolates obtained from any individual country varied from 65% to 82%. Most of the main subclusters within molecular type VNI also contained isolates from different countries, indicating gene flow and strain dispersal between South American countries, Spain, or both. However, all isolates could be separated by their unique PCR-fingerprinting pattern, with the highest homology being 84% between two unrelated environmental isolates from Mexico City (LA 22, budgerigar [parakeet] droppings, and LA 25, pigeon droppings).

Of the 266 clinical isolates, 177 (66.5%) were obtained from HIV-positive patients, with 139 (78.5%) being VNI, 14 (7.9%) VNII, 13 (7.4%) VNIII, 6 (3.4%) VNIV, 3 (1.7%) VGII, and 2 (1.1%) VGIII. Most (86.4%) isolates from HIV-positive patients belonged to the molecular types VNI and VNII, representing serotype A, *C. neoformans* var. *grubii*. Of these, 266 clinical isolates, 51 (19.2%) were recovered from patients with no reported risk factors. From those, 23 (45.1%) were var. *grubii* with the molecular type VNI (n=21) or VNII (n=2), and 28 (54.9%) were var. *gattii* with the molecular types VGI (n=3), VGII (n=10), VGIII (n=14), and VGIV (n=1). For 26 of the clinical isolates, no data concerning risk factors were available. Six veterinary isolates were molecular type VGI, and one was VGII. Most of the environmental isolates belonged to *C. neoformans* var. *grubii* with 73.1% being VNI (n=49) and 1.5% VNIII (n=1) AD hybrids. The remaining 17 (25.3%) isolates were *C. neoformans* var. *gattii* with the molecular type VGI (n=1), VGII (n=3), VGIII (n=12), and VGIV (n=1).

Cryptococcal isolates included in the IberoAmerican study were more frequently obtained from men than from women. The male-to-female ratio was 2l1 to 41, i.e., cryptococcosis was 5.1 times more common in men than in women. In the HIV-positive population alone, the incidence of cryptococcosis was 5.5 times more frequent in men than in women, based on the data obtained from the isolates investigated in this study. The age of the patients with clinically manifested cryptococcosis ranged from 4 to 73; 175 (65.9%) were between 21 and 40 years old.

The clinical isolates submitted to this study were collected over a period of 41 years, 1961–2001; most (92.5%) of the isolates were collected in the mid-1990s. The veterinary isolates were recovered from goats in Spain in 1995 and from a parrot in Brazil in 2000. The environmental isolates submitted were collected over a period of 7 years, 1993–2000.

## Discussion

This retrospective study of cryptococcosis in IberoAmerica was set up in an effort to establish a network of medical mycology laboratories to study the distribution of cryptococcal isolates, including the varieties and molecular types within the participating countries. The network was aimed at generating PCR-fingerprint and *URA5* RFLP patterns under standardized conditions in the Molecular Mycology Laboratory of the University of Sydney at Westmead Hospital, for a subset of clinical and environmental *C. neoformans* isolates from each participating country. These reference profiles are now available to each participating laboratory so they can set up the molecular typing techniques in their own laboratories, and to serve as internal controls in future extended studies of cryptococcal isolates in each country.

The data were obtained from a random selection of cryptococcal isolates from each participating country and laboratory, which do not necessarily reflect the true situation in IberoAmerica. Nonetheless, the data offer a general overview of molecular types and variety distribution of *C. neoformans* in IberoAmerica. For the first time, two different molecular typing techniques, PCR-fingerprinting with the minisatellite specific primer M13 and *URA5* gene RFLP analysis, were applied simultaneously to the same set of cryptococcal isolates, demonstrating identical groupings to the eight major molecular types previously described (*14*,*18*).

Previous pilot studies that used PCR-fingerprinting at the University of Sydney at Westmead Hospital distributed more than 400 clinical and environmental isolates obtained from Argentina, Australia, Belgium, Brazil, Germany, Italy, New Zealand, Papua New Guinea, South Africa, Thailand, Uganda, and the United States in eight major molecular types, VNI and VNII (serotype A), VNIII (serotype AD), VNIV (serotype D), VGI, VGII, VGIII and VGIV (serotypes B and C) (*14*,*18*). At the time of this original work, the molecular types VNI and VGI were found to be the most common genotypes worldwide. The present study, which includes more isolates from Latin America, showed the same results as regards variety *grubii,* with VNI being the predominant molecular type, accounting for 68.2% of all isolates. However, the situation changed drastically for variety *gattii*; as in this study, the predominant molecular type was VGIII, accounting for 9.1% of all isolates, in contrast to previous studies which showed that the molecular type VGIII was geographically restricted to India and the United States (*18*,*19*). In the present study, VGIII was also found in Argentina, Colombia, Guatemala, Mexico and Venezuela, suggesting that it is not as limited as previously suggested. The same was true for the molecular type VGIV, previously assigned only to India and South Africa (*18*,*19*); its presence in Colombia and Mexico, although in very low numbers, indicates a wider geographic distribution.

In general, the most common variety was *C. neoformans* var. *grubii,* 73.8% (n=251), followed by variety *gattii,* 20.3% (n=69). Much less common were the AD hybrids, 4.1% (n=14) and variety *neoformans,* 1.8% (n=6), which reflects the global distribution previously established (*14*,*18*,*23*).

The overall grouping of the isolates into eight major molecular types by PCR-fingerprinting with the minisatellite specific primer M13, obtained in this study and the previous pilot study by Meyer et al. 1999 (*14*) and Ellis et al. 2000 (*18*), agrees with the findings by Boekhout et al. 2001 (*23*) and by Cogliati et al. in 2000 (*24*). Boekhout et al. (*23*) used AFLP analysis to study 206 global isolates of *C. neoformans*, and grouped them into six major AFLP groups, whereas Cogliati et al. (*24*), using a slightly modified PCR-fingerprinting technique with the microsatellite specific primer (GACA)_4,_ grouped Italian isolates of *C. neoformans* var. *grubii* and var. *neoformans* into four major molecular types. Comparable molecular/genotypes, where identified in the four cited independent studies, are VNI = AFLP1 = Cogliati VN6 (serotype A, var. *grubii*); VNII = AFLP1A (serotype A, var. *grubii*); VNIII = AFLP3 = Cogliati VN3 and VN4 (serotype AD, hybrid between var. *grubii* and var. *neoformans*); VNIV = AFLP2 = Cogliati VN1 (serotype D, var. *neoformans*); VGI = AFLP4, VGII = AFLP6, VGIII = AFLP5 and VGIV (all corresponding to serotypes B and C, var. *gattii*) (*14*,*18*,*23*,*24*).

The overall results show some clonality between isolates obtained from a certain country or even between different countries, suggesting partial clonal spread of the pathogenic yeast within South America. However, the approximately 50%, overall similarity between *C. grubii* isolates, with the highest being 82%, suggests that these South American isolates are more varied than those obtained in a previous study by Franzot et al. (*25*), in which they examined a limited number of isolates from Brazil by using less discriminatory molecular techniques (CNRE-1 RFLP analysis and *URA5* sequencing). In Franzot’s study, the highest similarity was >94% between the Brazilian isolates, suggesting a higher clonality than observed in the isolates obtained from New York City studied in the same paper (*25*).

Interestingly, Chile and Spain share similar molecular types. Both countries have a large number of molecular type VNIII isolates (AD hybrids), 15.8% and 42.1%, respectively, although VNIV serotype D isolates were present only in Chile (26.3%). These groups are usually common in a number of European countries, such as France and Italy (*26*–*28*). However, only these two countries show two different *URA5* RFLP patterns, one consisting of seven bands, indicating a hybrid between VNI, VNII, and VNIV, and a second new hybrid *URA5* RFLP pattern, consisting of six bands, indicating a hybrid between VNII and VNIV. As a result of the IberoAmerican study, these hybrid patterns had recently been reported as part of Jackson’s honor’s thesis work (Jackson and Meyer unpub. data, 2000). The seven-band *URA5* RFLP pattern was exclusively found in Spain (n=3) and Chile (n=2). These strains seem to be triploid, and cloning with subsequent sequencing of the PCR product showed that they contain three different copies of the *URA5* gene. The six-band *URA5* RFLP pattern found in Spain (n=5) and Chile (n=1), was also found in Mexico (n=1) and Argentina (n=2), possibly due to the presence of the molecular types VNII and VNIV in these countries (*14*). These hybrid isolates are diploid at the *URA5* locus and contain two different copies of the gene (Jackson and Meyer, unpub. data).

Further studies are needed to investigate the special relationship between isolates obtained from these two countries. The similarity in the molecular types obtained from Spanish and Chilean isolates provides further evidence, that the cryptococcal strains present today in South America could be introduced during the European colonization. This idea had been suggested by Franzot et al. (*25*) when investigating isolates obtained from Brazil. The authors argue that the pigeon (*Columba livia*), thought to provide a major reservoir of *C. neoformans* in pigeon excreta, is believed to have originated in southern Europe and northern Africa and has been dispersed worldwide by human travel (*29*).

Most of the cryptococcal isolates in this study were recovered from patients whose main risk factor was HIV infection. Overwhelming numbers of these isolates corresponded to the molecular type VNI, in accordance with previous findings, showing that isolates of this molecular type are the major source of infection in HIV-positive patients worldwide (*18*,*19*). This finding highlights the fact that most human cryptococcal infections are caused by *C. neoformans* var. *grubii,* serotype A (*4*,*5*). A distinct picture emerged in the group of isolates obtained from patients with no known risk factors, as most were *C. neoformans* var. *gattii* isolates (n=28), with the molecular types VGI (n=3), VGII (n=10), VGIII (n=14), and VGIV (n=1), compared to 23 isolates belonging to the overall most common molecular type, VNI (41.2%) of *C. neoformans* var. *grubii*. This finding supports the conclusion that variety *gattii* primarily infects immunocompetent patients as Chen et al. had found when investigating Australian isolates (*30*). These authors have proposed that aboriginal people living in rural areas of Australia’s Northern Territory have a higher risk of cryptococcosis because they live in close proximity to the potential natural host of *C. neoformans* var. *gattii,* the eucalyptus trees (*30*).

Despite the fact that isolates included in this study constituted a random sampling, the results show again that HIV infection is the most important risk factor for cryptococcosis (*31*). This conclusion is supported by the number of isolates recovered from HIV-positive patients (n=177), the age distribution, which peaks between 20 and 40 years of age, and the date of isolation with a peak corresponding to the 1990s.

Overall, the network of mycology laboratories established in IberoAmerica provided, for the first time, a baseline knowledge of *C. neoformans* variety and molecular type distribution in the participating countries, placing the IberoAmerican isolates in the global picture of cryptococcosis.

## Supplementary Material

Appendix Table 1 Cryptococcus neoformans clinical, human, and veterinary isolates studied by country, institution characteristics, variety (serotype), and molecular type as identified by PCR fingerprinting with the primer M13 and URA5 RFLP analysisª

Appendix Table 2 Cryptococcus neoformans environmental isolates studied by country, institution characteristics, variety (serotype) and molecular type as identified by PCR-fingerprinting with the primer M13 and URA5 RFLP analysisª

## References

[1] Kwon-Chung KJ. A new genus, *Filobasidiella*, the perfect state of *Cryptococcus neoformans.* Mycologia. 1975;67:1197–200. 10.2307/3758842 765816

[2] Kwon-Chung KJ. A new genus, *Filobasidiella*, the sexual state of *Cryptococcus neoformans* B and C serotypes. Mycologia. 1976;68:942–6. 10.2307/3758813 790173

[3] Franzot SP, Salkin IF, Casadevall A. *Cryptococcus neoformans* var. *grubii*: separate varietal status for *Cryptococcus neoformans* serotype A isolates. J Clin Microbiol. 1999;37:838–40. 9986871 10.1128/jcm.37.3.838-840.1999 PMC84578

[4] Casadevall A, Perfect JR. *Cryptococcus neoformans*. Washington: ASM Press; 1998.

[5] Mitchell TG, Perfect JR. Cryptococcosis in the era of AIDS—100 years after the discovery of *Cryptococcus neoformans.* Clin Microbiol Rev. 1995;8:515–48. 8665468 10.1128/cmr.8.4.515 PMC172874

[6] Lazera MS, Pires FDA, Camillo-Coura L, Nishikawa MM, Bezerra CCF, Trilles L, Natural habitat of *Cryptococcus neoformans* var. *neoformans* in decaying wood forming hollows in living trees. J Med Vet Mycol. 1996;34:127–31. 10.1080/02681219680000191 8732358

[7] Ellis DH, Pfeiffer TC. Natural habitat of *Cryptococcus neoformans* var. *gattii.* J Clin Microbiol. 1990;28:1642–4. 2199524 10.1128/jcm.28.7.1642-1644.1990 PMC268004

[8] Sorrell TC. *Cryptococcus neoformans* variety *gattii.* Med Mycol. 2001;39:155–68. 11346263

[9] Lazera M, Cavalcanti M, Trilles L, Nishikawa M, Wanke B. *Cryptococcus neoformans* var. *gattii*—evidence for a natural habitat related to decaying wood in a pottery tree hollow. Med Mycol. 1998;36:112–9. 9776823

[10] Lazera MS, Salmito MA, Londero AT, Trilles L, Nishikawa M, Wanke B. Possible primary ecological niche of *Cryptococcus neoformans.* Med Mycol. 2000;38:379–83. 11092385 10.1080/mmy.38.5.379.383

[11] Callejas A, Ordóñez N, Rodríguez MC, Castañeda E. First isolation of *Cryptococcus neoformans* var. *gattii*, serotype C, from the environment in Colombia. Med Mycol. 1998;36:341–4. 10075505

[12] Brandt ME, Hutwagner LC, Kuykendall RJ, Pinner WS. Comparison of multilocus enzyme electrophoresis and random amplified polymorphic DNA analysis for molecular subtyping of *Cryptococcus neoformans.* J Clin Microbiol. 1995;33:1890–5. 7665665 10.1128/jcm.33.7.1890-1895.1995 PMC228292

[13] Crampin AC, Mathews RC, Hall D, Evans EG. PCR fingerprinting *Cryptococcus neoformans* by random amplification of polymorphic DNA. J Med Vet Mycol. 1993;31:463–5. 10.1080/02681219380000611

[14] Meyer W, Marszewska K, Amirmostofina M, Igreja RP, Hardtke C, Methling K, Molecular typing of global isolates of *Cryptococcus neoformans* var. *neoformans* by PCR-fingerprinting and RAPD. A pilot study to standardize techniques on which to base a detailed epidemiological survey. Electrophoresis. 1999;20:1790–9. 10.1002/(SICI)1522-2683(19990101)20:8<1790::AID-ELPS1790>3.0.CO;2-2 10435451

[15] Currie BP, Freundlich IF, Casadevall A. Restriction fragments length polymorphism analysis of *Cryptococcus neoformans* isolates from environmental (pigeons excreta) and clinical sources in New York City. J Clin Microbiol. 1994;32:1188–92. 7914203 10.1128/jcm.32.5.1188-1192.1994 PMC263640

[16] Spitzer SG, Spitzer ED. Characterization of the CNRE-1 family of repetitive the DNA elements in *Cryptococcus neoformans.* Gene. 1994;144:103–6. 10.1016/0378-1119(94)90211-9 8026743

[17] Varma A, Kwon-Chung KJ. DNA probes for strain typing of *Cryptococcus neoformans.* J Clin Microbiol. 1992;30:2960–7. 1452666 10.1128/jcm.30.11.2960-2967.1992 PMC270560

[18] Ellis D, Marriott D, Hajjeh RA, Warnock D, Meyer W, Barton R. Epidemiology: surveillance of fungal infections. Med Mycol. 2000;38:173–82. 11204143

[19] Meyer W, Kidd S, Castañeda A, Jackson S, Huynh M, Latouche GN, Global molecular epidemiology offers hints towards ongoing speciation within *Cryptococcus neoformans*. In: Abstracts of the 5th International Conference on *Cryptococcus* and Cryptococcosis, Adelaide, Australia, March 3–7, 2002. Adelaide: South Australian Postgraduate Medical Education Association; 2002.

[20] Kwon-Chung KJ, Bennet JE. Medical mycology. Philadelphia: Lea & Febiger Press; 1992. p. 397–446.

[21] Kwon-Chung KJ, Polacheck I, Bennet JE. Improved diagnostic medium for separation of *Cryptococcus neoformans* var. *neoformans* (serotype A and D) and *Cryptococcus neoformans* var. *gattii* (serotype B and C). J Clin Microbiol. 1982;15:535–7. 7042750 10.1128/jcm.15.3.535-537.1982 PMC272134

[22] Vassart G, Georges M, Monsieur R, Brocas H, Lequarre AN, Christophe D. A sequence in M13 phage detects hypervariable minisatellites in human and animal DNA. Science. 1986;246:683–4. 10.1126/science.2880398 2880398

[23] Boekhout T, Theelen B, Diaz M, Fell JW, Hop WCJ, Abeln ECA, Hybrid genotypes in the pathogenic yeast *Cryptococcus neoformans.* Microbiology. 2001;147:891–907. 11283285 10.1099/00221287-147-4-891

[24] Cogliati M, Allaria M, Liberi G, Tortorano AM, Viviani MA. Sequence analysis and ploidy determination of *Cryptococcus neoformans.* J Mycol Med. 2000;10:171–6.

[25] Franzot SP, Hamdan JS, Currie BP, Casadevall A. Molecular epidemiology of *Cryptococcus neoformans* in Brazil and the United States: evidence for both local genetic differences and a global clonal population structure. J Clin Microbiol. 1997;35:2243–51. 9276395 10.1128/jcm.35.9.2243-2251.1997 PMC229947

[26] Drommer F, Mathoulin S, Dupont B, Laporte A. Epidemiology of cryptococcosis in France: a 9 year survey (1985–1993). Clin Infect Dis. 1996;23:82–90. 8816134 10.1093/clinids/23.1.82

[27] Viviani MA, Wen H, Roverselli A, Calderelli-Stefano R, Cogliati M, Ferrante P, Identification by polymerase chain reaction fingerprinting of *Cryptococcus neoformans* serotype AD. J Med Vet Mycol. 1997;35:355–60. 10.1080/02681219780001411 9402529

[28] Tortorano AM, Viviani MA, Rigoni AL, Cogliati M, Roverselli A, Pagano A. Prevalence of serotype D in *Cryptococcus neoformans* isolates from HIV positive and HIV negative patients in Italy. Mycoses. 1997;40:297–302. 10.1111/j.1439-0507.1997.tb00235.x 9476513

[29] Johnston RF. Birds of North America. no 13. Philadelphia: The American Ornithologists Union and the Academy of natural Sciences of Philadelphia; 1992.

[30] Chen S, Sorrell T, Nimmo G, Speed B, Currie BJ, Marriott D, Epidemiology and host- and variety-dependent characterisation of infection due to *Cryptococcus neoformans* in Australia and New Zealand. Clin Infect Dis. 2000;31:499–508. 10.1086/313992 10987712

[31] Hajjeh RA, Conn LA, Stephens DS Baughman W, Hamill R, Graviss E, et al. Cryptococcosis: population-based multistate active surveillance and risk factors in human immunodeficiency virus-infected persons. J Infect Dis. 1999;179:449–54. 10.1086/314606 9878030

