# Supplementary material for: Molecular Typing of IberoAmerican Cryptococcus neoformans Isolates
Source: Emerg Infect Dis. 2003 Feb;9(2):189–95. doi: 10.3201/eid0902.020246 (PMC2901947; doi:10.3201/eid0902.020246)
Supplement: Appendix Table 1 — Cryptococcus neoformans clinical, human, and veterinary isolates studied by country, institution characteristics, variety (serotype), and molecular type as identified by PCR fingerprinting with the primer M13 and URA5 RFLP analysisª [file 02-0246_appT1-s1.pdf]

Appendix Table 1. *Cryptococcus neoformans* clinical, human, and veterinary isolates studied by country, institution characteristics, variety (serotype), and molecular type as identified by PCR fingerprinting with the primer M13 and *URA5* RFLP analysis[a]

LA code	Original code	Year	Original city	Sample	Gender	Age	Risk factor	Variety (serotype)	Molecularar type
Argentina									
Hospital Muñiz, Buenos Aires—Human Isolates									
LA 286#	135773	1999	Tucumán	CSF	F	25	HIV+	*grubii*	VNI
LA 28#	135868	1999	Buenos Aires	CSF	F	24	HIV+	*grubii*	VNI
LA 288#	136725	1999	Buenos Aires	CSF	M	31	HIV+	*grubii*	VNI
LA 289#	137235	1999	Buenos Aires	Blood	F	36	HIV+	*grubii*	VNI
LA 290#	136353	1999		CSF	M	50	PCM	*gattii*	VGIII
LA 291#	137965	1999	Córdoba	Urine	M	64	Mielodysplasia	*grubii*	VNI
LA 292#	144408	2000	Buenos Aires	Blood	M	28	HIV+	*grubii*	VNI
LA 293#	146323	2000	Buenos Aires	Blood	M	28	HIV+	*grubii*	VNI
LA 294#	146783	2000	Buenos Aires	CSF	M	28	HIV+	*grubii*	VNI
LA 295	143839	2000	Chaco	CSF	M	40	none	*gattii*	VGII
LA 296#	139856	1999	Buenos Aires	Node	M	35	HIV+	*grubii*	VNI
LA 297#	142181	1999	Buenos Aires	Blood	M	31	HIV+	*grubii*	VNI
LA 298#	136105	1999	Peru-Buenos Aires	Blood	M	40	HIV+	*grubii*	VNII
LA 299#	138493	1999	Buenos Aires	Urine	M	30	HIV+	*grubii*	VNI
LA 300#	138925	1999	Buenos Aires	CSF	M	31	HIV+	*grubii*	VNI
LA 301#	137154	1999	Buenos Aires	Bone marrow	M	37	HIV+	*grubii*	VNI
LA 302#	137141	1999	Buenos Aires	Blood	M	37	HIV+	*grubii*	VNI
LA 303#	137182	1999	Buenos Aires	Blood	M	25	HIV+	*grubii*	VNI
LA 304#	137921	1999	Buenos Aires	CSF	M	25	HIV+	*grubii*	VNI
LA 305#	145554	1999	Buenos Aires	Blood	M	32	HIV+	*grubii*	VNI
LA 306#	145535	1999	Uruguay- Buenos Aires	CSF	M	35	HIV+	*grubii*	VNI
LA 307#	145469	1999	Buenos Aires	Skin	M	49	transplant	*grubii*	VNI
LA 308#	145516	1999	Buenos Aires	Blood	M	30	HIV+	*grubii*	VNI
LA 309#	137278	1999	Adrogue	CSF	M	20	HIV+	*grubii*	VNI
LA 310#	140086	1999	Buenos Aires	CSF	F	29	HIV+	*grubii*	VNI
LA 311#	135610	1999	Buenos Aires	CSF	M	33	HIV+	*Hybrid*	VNIII*
LA 312#	138860	1999	Buenos Aires	CSF	F	31	HIV+	*grubii*	VNI
LA 313#	138205	1999	Buenos Aires	CSF	M	37	HIV+	*grubii*	VNI
LA 314#	138984	1999	Pergamino	CSF	M	23	HIV+	*grubii*	VNI
LA 315#	140102	1999	Buenos Aires	CSF	M	26	HIV+	*grubii*	VNI
LA 316#	137939	1999	Buenos Aires	CSF	F	34	HIV+	*grubii*	VNI
LA 318#	138027	1999	Buenos Aires	BAL	M	23	none	*grubii*	VNI
LA 319#	144687	2000	Buenos Aires	CSF	F	51	HIV+	*grubii*	VNI
LA 320#	144603	2000	Buenos Aires	Skin	F	50	HIV+	*grubii*	VNI
LA 321#	137332	1999	Buenos Aires	CSF	M	55	HIV+	*Hybrid*	VNIII*
LA 322#	135988	1999	Buenos Aires	Blood	M	54	HIV+	*grubii*	VNI
LA 323#	135887	1999	Buenos Aires	Blood	F	46	HIV+	*grubii*	VNI
LA 324#	136639	1999	Buenos Aires	CSF	M	29	HIV+	*grubii*	VNII
Departamento Micología, INEI, ANLIS “Dr C.G. Malbran,” Buenos Aires—Human Isolates									
LA 423#	00-381	2000	Buenos Aires	BAL	M	32	ISP	*grubii*	VNI

LA 424#	00-345	2000	Jujuy	CSF			HIV+	*grubii*	VNI
LA 425#	00-343	2000	Buenos Aires	CSF	M	58	ND	*grubii*	VNII
LA 426#	00-284	2000	Misiones	CSF	M	32	HIV+	*grubii*	VNI
LA 427#	00-236	2000	Mendoza	CSF			HIV+	*grubii*	VNI
LA 428#	00-364	2000	Buenos Aires	CSF	M	20	HIV+	*grubii*	VNII
LA 429#	962279	1996	Buenos Aires	CSF			HIV+	*grubii*	VNI
LA 430	962278	1996	Buenos Aires	CSF			HIV+	*grubii*	VNI
LA 431#	961935	1996	Buenos Aires	CSF			HIV+	*grubii*	VNI
LA 432#	972724	1998	Buenos Aires	CSF			HIV+	*grubii*	VNI
LA 433#	972756	1997	Buenos Aires	CSF	M	35	HIV+	*grubii*	VNI
LA 434#	982808	1998	Buenos Aires	CSF	M	30	HIV+	*grubii*	VNI
LA 435#	983036	1998	Buenos Aires	CSF	M		HIV+	*grubii*	VNI
LA 436#	983039	1998	Buenos Aires	CSF	M		HIV+	*grubii*	VNII
LA 437#	993126	1999	Buenos Aires	CSF	M	31	HIV+	*grubii*	VNI
Brazil									
Fundaçao Oswaldo Cruz, Rio de Janeiro—Human Isolates									
LA 55#	417	1995	Piaui State	CSF	M			*gattii*	VGII
LA 57	506	1995	Piaui State	CSF	M			*gattii*	VGII
LA 58#	512	1995	Piaui State	CSF	F			*grubii*	VNI
LA 59#	513	1995	Piaui State	CSF	M			*grubii*	VNI
LA 61#	557	1997	Piaui State	CSF	F		HIV+	*gattii*	VGII
LA 63#	567	1997	Piaui State	CSF	M			*grubii*	VNI
Universidade Estadual Paulista, Araraquara—Human Isolates									
LA 346#	481(5)	1995	São José do Rio Preto	CSF	F	24	HIV+	*grubii* (A)	VNI
LA 347#	482(5)	1995	São José do Rio Preto	CSF	F	24	HIV+	*grubii* (A)	VNI
LA 348	540(9)	1995	São José do Rio Preto	CSF	M	26	HIV+	*grubii* (A)	VNI
LA 349#	541(9)	1995	São José do Rio Preto	CSF	M	26	HIV+	*grubii* (A)	VNI
LA 350#	14(9)	1996	São José do Rio Preto	CSF	M	26	HIV+	*grubii* (A)	VNI
LA 351	13(11)	1996	São José do Rio Preto	CSF	M	26	HIV+	*grubii* (A)	VNI
LA 352#	49(11)	1996	São José do Rio Preto	CSF	M	26	HIV+	*grubii* (A)	VNI
LA 353#	98(17)	1996	São José do Rio Preto	CSF	M	40	HIV+	*grubii* (A)	VNI
LA 354#	14(17)	1996	São José do Rio Preto	CSF	M	40	HIV+	*grubii* (A)	VNI
LA 355#	221(23)	1996	São José do Rio Preto	CSF	M	42	HIV+	*grubii* (A)	VNI
LA 356#	18(23)	1997	São José do Rio Preto	CSF	M	42	HIV+	*grubii* (A)	VNI
LA 357#	222(24)	1996	São José do Rio Preto	CSF	M	30	HIV+	*grubii* (A)	VNI
LA 358	65(24)	1997	São José do Rio Preto	CSF	M	22	HIV+	*grubii* (A)	VNI
LA 359#	62(27)	1997	São José do Rio Preto	CSF	M	29	HIV+	*grubii* (A)	VNI
LA 360#	66(27)	1997	São José do Rio Preto	CSF	M	29	HIV+	*grubii* (A)	VNI
LA 361#	67(27)	1997	São José do Rio Preto	CSF	M	30	HIV+	*grubii* (A)	VNI
LA 363#	1342	1993	Araraquara	CSF	M	27	HIV+	*grubii* (A)	VNI
LA 364#	1755	1997	Araraquara	CSF	F	20	HIV+	*grubii* (A)	VNI
LA 365#	1920	1998	Araraquara	CSF	M	64	HIV+	*grubii* (A)	VNI
LA 366#	2177	2000	Araraquara	CSF	M	50	HIV+	*grubii* (A)	VNI
Universidade Estadual Paulista, Araraquara - Veterinary Isolates									
LA 362 #	Bird1	2000	Jaboticabal	Parrot liver				*gattii* (B)	VGII
Instituto Adolfo Lutz, São Paulo—Human Isolates									
LA 32#	LCS-534		São Paulo	CSF	M	30	HIV+	*grubii*	VNI
LA 328#	GFR-1505	1997	São Paulo	CSF	F	30	HIV+	*grubii*	VNI

LA 329#	AFG-34	2000	São Paulo	CSF	M	29	HIV+	*grubii*	VNI
LA 330#	AFG-14	2000	São Paulo	CSF	M	30	HIV+	*grubii*	VNI
LA 331#	CMS-1226	1996	São Paulo	CSF	M	28	HIV+	*grubii*	VNI
LA 332#	AFG-25/00	2000	São Paulo	CSF	M	35	HIV+	*grubii*	VNI
LA 333#	PRL-1133	1996	São Paulo	CSF	M	40	HIV+	*grubii*	VNI
LA 334#	JCJ-1157	1996	São Paulo	CSF	M	32	HIV+	*grubii*	VNI
LA 335#	MAAC-1866	1996	São Paulo	CSF	M	36	HIV+	*grubii*	VNI
LA 336#	VPD-25	1996	São Paulo	CSF	F	28	HIV+	*grubii*	VNI
LA 337#	AFG-09/00	1996	São Paulo	CSF	M	30	HIV+	*gattii*	VGII
LA 338#	AFG-15/00	2000	São Paulo	CSF	M	30	HIV+	*gattii*	VGII
LA 339#	LCS-394	1996	São Paulo	CSF	M	31	HIV+	*grubii*	VNI
LA 340#	IFOC-1446	1996	São Paulo	CSF	M	36	HIV+	*grubii*	VNI
LA 341#	MO-438	1996	São Paulo	CSF	M	34	HIV+	*grubii*	VNI
LA 342#	KSO-948	1996	São Paulo	CSF	M	23	HIV+	*grubii*	VNI
LA 343#	CSC-1016	1996	São Paulo	CSF	M	34	HIV+	*grubii*	VNII
LA 344#	MDS-949	1996	São Paulo	CSF	M	32	HIV+	*grubii*	VNI
LA 345#	MEM-1064	1996	São Paulo	CSF	M	33	HIV+	*grubii*	VNI
Centro de Biotechnologia, Porto Alegre—Human Isolates									
LA 755	HC2	2000	Porto Alegre	CSF	M	38	HIV+	*grubii* (A)	VNI
LA 756	HC3	2000	Porto Alegre	CSF	M	38	HIV+	*grubii* (A)	VNII
LA 757	HC4	2000	Porto Alegre	CSF	F	36	HIV+	*grubii* (A)	VNI
LA 758	HC6	2000	Porto Alegre	CSF	M	46	HIV+	*grubii* (A)	VNI
LA 759	HC8	2000	Porto Alegre	CSF	M	40	HIV+	*grubii* (A)	VNI
LA 760	HC9	2000	Porto Alegre	CSF	M	33	HIV+	*grubii* (A)	VNI
LA 761	HC10	2000	Porto Alegre	CSF	M	29	HIV+	*grubii* (A)	VNI
LA 762	HC11	2000	Porto Alegre	CSF	F	50	HIV+	*grubii* (A)	VNI
LA 763	HC13	2000	Porto Alegre	CSF	M	42	HIV+	*grubii* (A)	VNI
LA 764	HC14	2000	Porto Alegre	CSF	M	29	HIV+	*grubii* (A)	VNI
LA 765	HC15	2000	Porto Alegre	CSF	F	55	HIV+	*grubii* (A)	VNI
Chile									
Universidad de Chile, Santiago de Chile—Human Isolates									
LA 256#	CR-01	1990	Santiago	Blood	M		HIV+	*neoformans*	VNIV
LA 258	CR-03	1991	Santiago	CSF	M		HIV+	Hybrid	VNIII*
LA 259#	CR-06	1991	Santiago	Blood	M		HIV+	*neoformans*	VNIV
LA 260	CR-10	1995	Santiago	Blood	M		HIV+	Hybrid	VNIII
LA 262#	CR-12	1997	Santiago	Blood	M	33	HIV+	*neoformans*	VNIV
LA 263	CR-16	1998	Santiago	CSF	M		HIV+	Hybrid	VNIII
LA 264#	CR-21	1997	Santiago	Pleural fluid	M		ND	*grubii*	VNI
LA 267#	CR-26	2000	Santiago	Abscess	M		HIV+	*grubii*	VNII
LA 268#	CR-27	2000	Santiago	Blood	M		HIV+	*neoformans*	VNIV
LA 269#	CR-29	2000	Santiago	BAL	M	57	HIV+	*grubii*	VNI
LA 270#	CR-30	2000	Santiago	Blood	M		HIV+	*grubii*	VNI
LA 271#	CR-31	2000	Santiago	Blood	M		HIV+	*grubii*	VNI
LA 272#	CR-34	2000	Santiago	CSF	M	29	HIV+	*neoformans*	VNIV
LA 273#	CR-35	2000	Viña del Mar	CSF	M	35	HIV+	*grubii*	VNII
LA 274#	CR-36	2000	Santiago	CSF	M		ND	*grubii*	VNII
Colombia									
Instituto Nacional de Salud, Bogotá—Human Isolates									

LA 214#	H0058-I-138	1991	Tolima	CSF	F	4	None	*grubii* (A)	VNI
LA 461#	H0058-I-34	1986	Casanare	CSF	F	16	None	*gattii* (B)	VGII
LA 469#	H0058-I-43	1987	Cundinamarca	CSF	M	67	ND	*grubii* (A)	VNII
LA 472#	H0058-I-50	1988	Antioquia	CSF	F		HIV+	*grubii* (A)	VNI
LA 473#	H0058-I-57	1988	Bogotá	CSF	M	58	None	*grubii* (A)	VNI
LA 478#	H0058-I-62	1988	Bogotá	CSF	F	16	Lupus	*grubii* (A)	VNI
LA 479#	H0058-I-64	1988	Huila	CSF	M	33	None	*grubii* (A)	VNI
LA 481#	H0058-I-67	1989	N. Santander	CSF	M	46	None	*grubii* (A)	VNI
LA 487#	H0058-I-78	1989	Arauca	CSF	M	43	None	*gattii* (C)	VGIII
LA 495	H0058-I-96	1990	Cundinamarca	CSF	M		None	*grubii* (A)	VNI
LA 498	H0058-I-103	1990	Antioquia	CSF	M	65	None	*grubii* (A)	VNI
LA 499#	H0058-I-106	1990	N. Santander	CSF	M	44	None	*gattii* (B)	VGII
LA 506#	H0058-I-160	1992	Tolima	CSF	M	28	None	*grubii* (A)	VNI
LA 511	H0058-I-190	1992	Bogotá	CSF	M	34	HIV+	*grubii* (A)	VNII
LA 516#	H0058-I-212	1993	N. Santander	CSF	M	54	None	*gattii* (B)	VGII
LA 518#	H0058-I-229	1993	Bogotá	CSF	M	33	HIV+	*grubii* (A)	VNI
LA 521#	H0058-I-232	1993	Bogotá	CSF	M	30	HIV+	*grubii* (A)	VNI
LA 523#	H0058-I-238	1994	Bogotá	CSF	M	42	HIV+	*grubii* (A)	VNI
LA 525	H0058-I-246	1994	Bogotá	CSF	M	65	None	*grubii* (A)	VNI
LA 550#	H0058-I-471	1996	Nariño	CSF	M	35	HIV+	*grubii* (A)	VNI
LA 552#	H0058-I-473	1997	Bogotá	CSF	M	40	HIV+	*grubii* (A)	VNI
LA 564	H0058-I-628	1997	Bogotá	CSF	M	29	None	*gattii* (B)	VGI
LA 567#	H0058-I-638	1997	Caquetá	CSF	F	34	None	*gattii* (B)	VGII
LA 568	H0058-I-642	1997	Boyacá	CSF	M	36	None	*gattii* (B)	VGIV
LA 572#	H0058-I-653	1998	Cundinamarca	CSF	M	57	HIV+	*grubii* (A)	VNII
LA 584#	H0058-I-675	1998	Bolivar	CSF	M	23	None	*gattii* (B)	VGII
LA 590#	H0058-I-762	1998	Santander	CSF	M	48	None	*gattii* (B)	VGII
LA 593#	H0058-I-789	1998	Bogotá	CSF	M	23	HIV+	*neoformans* (D)	VNIV
LA 595#	H0058-I-828	1998	Bogotá	CSF	M	52	HIV+	*grubii* (A)	VNII
LA 599#	H0058-I-881	1999	N. Santander	CSF	M	33	None	*gattii* (B)	VGII
LA 602#	H0058-I-889	1999	Bogotá	CSF	M	42	HIV+	*grubii* (A)	VNII
LA 603#	H0058-I-902	1999	Arauca	CSF	M	50	None	*grubii* (A)	VNII
LA 615#	H0058-I-1068	2000	Antioquia	CSF	M	33	HIV+	*grubii* (A)	VNI
LA 620	H0058-I-1097	2000	Antioquia	BAL	M	19	HIV+	*grubii* (A)	VNII
LA 622#	H0058-I-1134	2000	Risaralda	CSF	F	41	ND	*gattii* (B)	VGIII
LA 628#	H0058-I-1198	2001	Bogotá	CSF	M	23	HIV+	*grubii* (A)	VNI
LA 629#	H0058-I-1199	2001	Bogotá	CSF	M	43	HIV+	*grubii* (A)	VNI
LA 752	H0058-I-1276	2001	N. Santander	CSF	M	29	None	*gattii* (C)	VGIII
LA 753#	H0058-I-1278	2001	N. Santander	CSF	M	39	None	*gattii* (B)	VGII
Guatemala									
Hospital San Juan de Dios and Universidad de San Carlos, Guatemala—Human Isolates									
LA 737#	134	2000	Guatemala City	CSF	F		HIV+	*grubii* (A)	VNI
LA 738#	188	2000	Guatemala City	CSF	M	57	HIV+	*grubii* (A)	VNI
LA 739#	415	2001	Guatemala City	Blood	M		ND	*grubii* (A)	VNI
LA 740#	600	2000	Guatemala City	CSF	M	53	HIV+	*grubii* (A)	VNI
LA 741	C.9							*grubii* (A)	VNI
LA 742#	C.9.4.67.BM(1)							*grubii* (A)	VNI
LA 743	C.9.7.17.G2(1)							*grubii* (A)	VNI

LA 744	C.9.98RM3-6							*grubii* (A)	VNI
LA 745	C.9.10.11.BG(2)							*grubii* (A)	VNI
LA 746	C.9.11.60AG1(1)							*grubii* (A)	VNI
LA 747	2591	2000		CSF	M		ND	*grubii* (A)	VNI
LA 748	3308	2000		CSF	M		ND	*grubii* (A)	VNI
LA 749#	3417	2000					ND	*grubii* (A)	VNI
LA 750#	2361	2000			F	14	ND	*gattii*	VGIII
LA 751#	193	2000		CSF	M	57	HIV+	*grubii* (A)	VNI
Mexico									
Universidad Nacional Autonoma de Mexico, Mexico City—Human Isolates									
LA 1#	116	1994	Mexico City	CSF	M	19	None	*gattii* (B)	VGI
LA 2#	117	1994	Mexico City	CSF	M	35	Tumor	*gattii* (B)	VGI
LA 3	122	1986	Mexico City	CSF	F	10	None	*gattii* (B)	VGIII
LA 4#	174	1996	Texcoco, Edo	CSF	F	15	IMS	*grubii*	VNI
LA 5	190	1995	Mexico City	CSF	F	45	None	*gattii*	VGIII
LA 6#	194	1995	Mexico City	CSF	M	27	HIV+	*grubii*	VNI
LA 7#	196	1995	Mexico City	CSF	M	27	HIV+	*grubii*	VNI
LA 8#	202	1996	Mexico City	CSF	M	38	HIV+	*grubii*	VNI
LA 9#	204	1996	Mexico City	CSF	M	39	None	*gattii*	VGI
LA 10	205	1996	Mexico City	CSF	M	67	None	*grubii*	VNI
LA 11#	207	1996	Mexico City	CSF	M	33	HIV+	*grubii*	VNI
LA 12#	209	1996	Mexico City	CSF	M	35	IMS	*grubii*	VNI
LA 13#	216	1996	Mexico City	CSF	M	46	None	*grubii*	VNI
LA 14#	218	1997	Mexico City	CSF	M	47	HIV+	Hybrid	VNIII*
LA 15#	220	1997	Mexico City	CSF	M	34	HIV+	*grubii*	VNI
LA 16#	221	1996	Mexico City	CSF	F	17	None	*gattii*	VGIII
LA 17#	223	1996	Mexico City	CSF	M	27	HIV+	*grubii*	VNI
LA 18#	224	1996	Mexico City	CSF	M	31	HIV+	*gattii*	VGIII
LA 19	225	1997	Mexico City	Blood	M	29	HIV+	*grubii*	VNI
LA 20	227	1997	Mexico City	CSF	M	36	HIV+	*gattii*	VGIII
Instituto Nacional de Diagnostica y Referencia Epidemiologicos, Mexico City—Human Isolates									
LA 390#	5604	1961	Distrito Federal	CSF	M	38	ND	*gattii*	VGIV
LA 391#	5605	1962	Distrito Federal	CSF	M	47	ND	*gattii*	VGIV
LA 392#	5606	1965	Distrito Federal	CSF	M	40	ND	*gattii*	VGIV
LA 393	5607	1965	Distrito Federal	CSF	M	52	ND	*grubii*	VNI
LA 394#	5608	1967	Distrito Federal	CSF	M	41	ND	*grubii*	VNI
LA 396#	5611	1970	Distrito Federal	CSF	M	33	ND	*grubii*	VNI
LA 397#	5613	1973	Distrito Federal	CSF	M	47	ND	*grubii*	VNI
LA 398#	5615	1978	Distrito Federal	Skin	M	59	ND	*grubii*	VNI
LA 399#	5616	1980	Distrito Federal	Lungs, CSF	M	59	ND	*gattii*	VGIII
LA 400#	5617	1980	Distrito Federal	CSF	F	32	ND	*gattii*	VGIII
LA 402#	5619	1982	Distrito Federal	CSF	M	35	ND	*gattii*	VGIII
LA 403	5620	1986	Distrito Federal	CSF	F	11	ND	*gattii*	VGIII
LA 404#	5621	1987	Michoacan	CSF	M	47	ND	*grubii*	VNII
LA 405#	5622	1988	Distrito Federal	CSF	M	43	ND	*grubii*	VNI
LA 406#	5623	1990	Distrito Federal	CSF	M	34	HIV+	*grubii*	VNI
LA 407	5624	1990	Distrito Federal	CSF	M	46	ND	*gattii*	VGIII
LA 408	5625	1991	Distrito Federal	Blood	M	49	ND	*grubii*	VNI

LA 409#	5626	1992	Distrito Federal	CSF	M	53	HIV+	*grubii*	VNI
LA 410#	5627	1992	Distrito Federal	CSF	M	37	HIV+	*grubii*	VNI
LA 411	5628	1991	Distrito Federal	CSF	M	29	ND	*gattii*	VGIII
LA 412#	5629	1997	Distrito Federal	CSF	M	58	ND	*gattii*	VGIII
LA 413#	5630	1998	Distrito Federal	CSF	M	30	HIV+	*grubii*	VNI
LA 414#	5631	1999	Distrito Federal	CSF	M	28	ND	*gattii*	VGIII
LA 415#	5632	1999	Distrito Federal	CSF	M	36	ND	*grubii*	VNI
LA 416#	5633	1999	Distrito Federal	CSF	M	42	HIV+	*grubii*	VNI
LA 417#	5634	1999	Distrito Federal	CSF	M	33	HIV+	*grubii*	VNI
Peru									
Instituto de Medicina Tropical Alexander von Humboldt, Lima—Human Isolates									
LA 153#	39401/U	1998	Lima	Urine	M	27	HIV+	*grubii*	VNI
LA 154#	39401/S	1998	Lima	Sputum	M	27	HIV+	*grubii*	VNI
LA 155#	39401/B	1998	Lima	Blood	M	27	HIV+	*grubii*	VNI
LA 156	39407/CSF	1998	Lima	CSF	M	30	HIV+	*grubii*	VNI
LA 157#	39407/B	1998	Lima	Blood	M	30	HIV+	*grubii*	VNI
LA 158#	39408/CSF	1998	Lima	CSF	M	22	HIV+	*grubii*	VNI
LA 159#	39408/U	1998	Lima	Urine	M	22	HIV+	*grubii*	VNI
LA 160#	39408/B	1998	Lima	Blood	M	22	HIV+	*grubii*	VNI
LA 162#	39410/U	1998	Lima	Urine	F	36	HIV+	*grubii*	VNI
LA 163#	39410/B	1998	Lima	Blood	F	36	HIV+	*grubii*	VNI
LA 164	39410/BM	1998	Lima	Blood marrow	F	36	HIV+	*grubii*	VNI
LA 169	8640	1988	Lima	Abscesses	M	66	None	*grubii*	VNI
LA 170#	15335	1993	Lima	CSF	M	36	None	*gattii*	VGI
Spain									
GREMEC, IMM/UAB, Barcelona—Human Isolates									
LA 180#	154c	1995	Barcelona	Blood	F		HIV+	Hybrid (A, AD)	VNIII
LA 181#	155c	1995	Barcelona	CSF	M		HIV+	Hybrid (A, AD)	VNIII
LA 182#	156c	1995	Barcelona	Blood	M		HIV+	*grubii* (A)	VNI
LA 183#	1c	1994	Valencia	CSF	M		HIV+	Hybrid (AD, D)	VNIII*
LA 184#	2c	1994	Valencia	CSF	F		HIV+	Hybrid (AD, D)	VNIII*
LA 185#	3c	1994	Valencia	CSF	M		HIV+	Hybrid (AD, D)	VNIII**
LA 186#	73c	1994	Sevilla	CSF	M		HIV+	Hybrid (AD)	VNIII
LA 187#	74c	1995	Sevilla	CSF	M		HIV+	*grubii* (A)	VNI
LA 188#	75c	1995	Sevilla	CSF	M		HIV+	Hybrid (AD)	VNIII*
GREMEC, IMM/UAB, Barcelona—Veterinary Isolates:									
LA 174#	48 A	1995	Cáceres	Goat				*gattii* (B)	VGI
LA 175#	50 A	1995	Cáceres	Goat				*gattii* (B)	VGI
LA 176#	52 A	1995	Cáceres	Goat				*gattii* (B)	VGI
LA 177#	54 A	1995	Cáceres	Goat				*gattii* (B)	VGI
LA 178#	56 A	1995	Cáceres	Goat				*gattii* (B)	VGI
LA 179#	58 A	1995	Cáceres	Goat				*gattii* (B)	VGI
Venezuela									
Universidad del Zulia, Maracaibo—Human Isolates									
LA 367#	BVC125-96	1996	Caracas	ND	F	36	HIV+	*grubii* (A)	VNII
LA 368#	BV1742	1996	Caracas	CSF	M	66	leukemia	*grubii* (A)	VNI
LA 369#	BV2086	1996	Caracas	CSF	M	34	HIV+	*grubii* (A)	VNI
LA 370#	BV2098	1996	Caracas	CSF	M	28	HIV+	*grubii* (A)	VNI

LA 371#	BV2189	1996	Caracas	CSF	M	26	HIV+	*grubii* (A)	VNI
LA 372#	BV2198	1996	Caracas	CSF	M	Adult	HIV+	*grubii* (A)	VNI
LA 373#	BV2199	1996	Caracas	CSF	M	34	HIV+	*grubii* (A)	VNI
LA 374#	BV2244	1997	Caracas	CSF	M	Adult	HIV+	*grubii* (A)	VNI
LA 375#	BV2293	1997	Caracas	CSF	M	Adult	HIV+	*grubii* (A)	VNI
LA 376#	BV2379	1997	Caracas	CSF	M	Adult	HIV+	*grubii* (A)	VNI
LA 377	BV2450	1997	Maracaibo	CSF	M	Adult	HIV+	*grubii* (A)	VNI
LA 379	BV1	1995	Maracaibo	CSF	M	28	HIV+	*grubii* (A)	VNI
LA 380	BV2	1995	Maracaibo	CSF	M	19	HIV+	*grubii* (A)	VNI
LA 381#	BV5	1996	Maracaibo	CSF	M	30	None	*gattii* (B)	VGII
LA 382#	BV19	1997	Maracaibo	CSF	M	32	None	*gattii* (C)	VGIII
LA 383#	BV201A	1997	Maracaibo	CSF	M	41	Transplant	*grubii* (A)	VNI
LA 384	BV170cli	1999	Maracaibo	CSF	M	73	None	*grubii*	VNI
LA 385#	BV1118	1999	Maracaibo	CSF	M	30	None	*grubii*	VNI
LA 386#	BV1218	1999	Maracaibo	CSF	F	42	MS	*gattii*	VGII
LA 387#	BV1297	1999	Maracaibo	CSF	F	25	Diabetes	*gattii*	VGII

[a]PCR, polymerase chain reaction; RFLP, restriction fragment length polymorphism; GREMEC, IMM/UAB,;CSF, cerebrospinal fluid; F, female; M, male; BAL, bronchoalveolar lavage; ISP, immunosupresed; MS, multiple sclerosis; ND, no data; VNIII, seven-band *URA5* RFLP pattern; VNIII*, six-band *URA5* RFLP pattern; *, strains included in the Gelcompar*II* analysis.
