# Supplementary material for: Molecular Typing of IberoAmerican Cryptococcus neoformans Isolates
Source: Emerg Infect Dis. 2003 Feb;9(2):189–95. doi: 10.3201/eid0902.020246 (PMC2901947; doi:10.3201/eid0902.020246)
Supplement: Appendix Table 2 — Cryptococcus neoformans environmental isolates studied by country, institution characteristics, variety (serotype) and molecular type as identified by PCR-fingerprinting with the primer M13 and URA5 RFLP analysisª [file 02-0246_appT2-s2.pdf]

Appendix Table 2. *Cryptococcus neoformans* environmental isolates studied by country, institution characteristics, variety (serotype) and molecular type as identified by PCR-fingerprinting with the primer M13 and *URA*5 RFLP analysis[a]

LA Code	Original code	Year	Original city	Sample	Variety (serotype)	Molecular type
Argentina						
Departamento Micología, INEI, ANLIS "Dr C.G. Malbran," Buenos Aires						
LA 438#	Cr34Eb	2000	Buenos Aires	Trees	grubii	VNI
LA 440#	CrA29	2000	Buenos Aires	Trees	*grubii*	VNI
LA 441#	Cr10	2000	Buenos Aires	Trees	*gattii*	VGI
LA 442#	CrA32	2000	Buenos Aires	Trees	*grubii*	VNI
Brazil						
Fundaçao Oswaldo Cruz, Rio de Janeiro						
LA 44#	414	1995	Piaui State	Pottery tree hollow	*gattii* (B)	VGII
LA 45#	415	1995	Piaui State	Pink shower tree hollow	*gattii* (B)	VGII
LA 47#	434	1996	Piaui State	*E. camaldulensis* debris	*gattii* (B)	VGII
LA 52#	561	1997	Piaui State	Pink shower tree hollow	*grubii*	VNI
LA 54	564	1997	Piaui State	Pink shower tree hollow	*grubii*	VNI
LA 419#	WM1012	2000	Rio de Janeiro	Pink shower tree 4, large hollow	*grubii*	VNI
LA 420#	WM1013	2000	Rio de Janeiro	Pink shower tree 4, large hollow	*grubii*	VNI
LA 421#	WM1014	2000	Rio de Janeiro	Pink shower tree 4, large hollow	*grubii*	VNI
LA 422#	WM1015	2000	Rio de Janeiro	Pink shower tree 4, large hollow	*grubii*	VNI
Chile						
Universidad de Chile, Santiago de Chile						
LA 276#	04-A	1993	Santiago	Bird droppings	*grubii*	VNI
LA 277#	06-A	1993	Santiago	Bird droppings	*grubii*	VNI
LA 281	21-A	1993	Santiago	Bird droppings	*grubii*	VNI
LA 284#	52-A	1993	Santiago	Bird droppings	*grubii*	VNI
Colombia						
Instituto Nacional de Salud, Bogota						
LA 235#	H0058-I-737	1998	N. Santander	Almond tree (9)	*gattii* (C)	VGIII
LA 241	H0058-I-1014	1999	N. Santander	Almond tree (20)	*gattii* (C)	VGIII
LA 631#	H0058-I-556	1997	N. Santander	Almond tree (9)	*gattii* (C)	VGIII
LA 645#	H0058-I-607	1997	N. Santander	Almond tree (9)	*grubii*	VNI
LA 651	H0058-I-613	1997	N. Santander	Almond tree (9)	*gattii* (C)	VGIII
LA 658#	H0058-I-679	1998	N. Santander	Almond tree (9)	*grubii* (A)	VNI
LA 659	H0058-I-682	1998	N. Santander	Almond tree (21)	*gattii* (C)	VGIII
LA 667	H0058-I-755	1998	N. Santander	Almond tree (9)	*gattii* (C)	VGIII
LA 672	H0058-I-798	1998	N. Santander	Almond tree (9)	*gattii* (C)	VGIV
LA 674	H0058-I-818	1998	N. Santander	Almond tree (9)	*gattii* (C)	VGIII
LA 676	H0058-I-820	1998	N. Santander	Almond tree (9)	*gattii* (C)	VGIII
LA 680	H0058-I-826	1998	N. Santander	Almond tree (9)	*gattii* (C)	VGIII
LA 684#	H0058-I-908	1998	N. Santander	Almond tree (9)	*gattii* (C)	VGIII
LA 688	H0058-I-548	1996	N. Santander	House patient 3	*grubii* (A)	VNI
LA 691#	H0058-I-551	1996	N. Santander	Older oiti tree	*grubii* (A)	VNI
LA 706#	H0058-I-587	1996	N. Santander	Oiti tree	*grubii* (A)	VNI
LA 710#	H0058-I-595	1996	N. Santander	Acacia tree (1)	*grubii* (A)	VNI

LA 714	H0058-I-599	1997	N. Santander	Acacia tree (6)	*gattii*	VGIII
LA 720#	H0058-I-735	1998	Bogotá	*Eucalyptus* tree (13)	*grubii* (A)	VNI
LA 726#	H0058-I-745	1997	N. Santander	Cuji tree	*grubii* (A)	VNI
LA 731	H0058-I-752	1997	N. Santander	Seed of almond tree	*grubii* (A)	VNI
LA 733#	H0058-I-757	1997	N. Santander	Cuji, tree	*gattii*	VGIII
LA 736	H0058-I-857	1998	Bogotá	*Eucalyptus* tree	*grubii* (A)	VNI
Mexico						
Universidad Nacional Autonoma de Mexico, Mexico City						
LA 21#	88	1994	Mexico City	Green parrot droppings	*grubii*	VNI
LA 22#	89	1994	Mexico City	Budgerigar droppings	*grubii*	VNI
LA 23#	90	1994	Mexico City	Budgerigar droppings	*grubii*	VNI
LA 24#	146	1995	Mexico City	Pigeon droppings	*grubii*	VNI
LA 25#	147	1995	Mexico City	Pigeon droppings	*grubii*	VNI
LA 26#	148 A	1995	Mexico City	Pigeon droppings	*grubii*	VNI
LA 27#	148 B	1995	Mexico City	Pigeon droppings	*grubii*	VNI
LA 28#	149	1995	Mexico City	Pigeon droppings	*grubii*	VNI
LA 29#	150 A	1995	Mexico City	Pigeon droppings	*grubii*	VNI
LA 30#	150 B	1995	Mexico City	Pigeon droppings	*grubii*	VNI
LA 31#	151	1995	Mexico City	Pigeon droppings	*grubii*	VNI
LA 32#	152	1995	Mexico City	Pigeon droppings	*grubii*	VNI
LA 33	164	1995	Mexico City	Pigeon droppings	*grubii*	VNI
LA 34#	165	1995	Mexico City	Pigeon droppings	*grubii*	VNI
LA 35#	168	1995	Mexico City	Pigeon droppings	*grubii*	VNI
LA 36#	170	1995	Mexico City	Pigeon droppings	*grubii*	VNI
LA 37#	230	1998	Edo, Mexico	*E. camaldulensis* (leaves and flowers)	*grubii*	VNI
LA 38#	234	1998	Edo, Mexico	*E. camaldulensis* (leaves and flowers)	*grubii*	VNI
LA 39#	235	1998	Edo, Mexico	*E. camaldulensis* (leaves and flowers)	*grubii*	VNI
LA 40#	237	1998	Edo, Mexico	*E. camaldulensis* (leaves and flowers)	*grubii*	VNI
LA 41#	238	1998	Edo, Mexico	*E. camaldulensis* (leaves and flowers)	*grubii*	VNI
LA 42	239	1998	Edo, Mexico	*E. camaldulensis* (leaves and flowers)	*grubii*	VNI
Instituto Nacional de Diagnostica y Referencia Epidemiologicos, Mexico City						
LA 418#	5635	2000	Distrito Federal	Pigeon excreta	*grubii*	VNI
Spain						
GREMEC, IMM/UAB, Barcelona						
LA 189 #	152 A	1999	Barcelona	Pigeon droppings	*grubii* (A)	VNI
LA 190 #	76 A	1999	Barcelona	Pigeon droppings	*grubii* (A)	VNI
LA 192 #	4 A	1997	Alacant	Pigeon droppings	Hybrid (A)(AD)	VNIII
LA 193 #	6 A	1997	Alacant	Pigeon droppings	*grubii* (A)	VNI

[a]RFLP, restriction fragment length polymorphism; INEI, Instituto Nacional de Enfermedades Infecciosas; ANLIS, Administracion Nacional de Laboratorios e Institutos de Salud; GREMEC, Grup de Recerca en Micologia Experimental i Clinica; IMM, Institut Municipal d' Investigacio Medica; UAB, Universita Autonoma de Barcelona.

[b]VNIII: seven-band *URA5* RFLP pattern;VNIII*, six-band *URA5* RFLP pattern; *, strains included in the Gelcompar*II* analysis
